# Hypertension in aortic coarctation

**DOI:** 10.3389/fcvm.2025.1505269

**Published:** 2025-04-07

**Authors:** Luisa Ye, Biagio Castaldi, Irene Cattapan, Alice Pozza, Jennifer Fumanelli, Giovanni Di Salvo

**Affiliations:** Pediatric Cardiology Unit, Department of Women’s and Children’s Health, University Hospital of Padua, Padua, Italy

**Keywords:** aortic coarctation, hypertension, hypertension in congenital heart diseases, congenital heart diseases, surgical repair, stent implantation

## Abstract

Aortic coarctation (AoC) is a common congenital heart defect, affecting 5%–8% of patients with structural congenital anomalies. Despite advances in surgical and percutaneous interventions, hypertension remains a significant complication in AoC patients, even after successful repair. Chronic hypertension develops in 20%–70% of patients and is a leading cause of long-term cardiovascular morbidity. In these patients, hypertension is associated to renin-angiotensin system activation, residual aortic arch abnormalities, and impaired aortic elasticity. Additionally, exercise-induced hypertension and masked hypertension contribute to adverse outcomes. Management of hypertension in AoC patients requires both perioperative and long-term care. Early after correction, intravenous antihypertensive agents, such as sodium nitroprusside, esmolol, and labetalol, are commonly used to stabilize blood pressure and reduce the risk of complications like cerebral hemorrhage. Oral beta-blockers, ACE inhibitors (ACE-Is), angiotensin receptor blockers (ARBs) and calcium channel blockers (CCBs) are most commonly used for chronic hypertension. In this review, we discussed about diagnostic workup and therapeutical strategies for hypertension in AoC patients.

## Introduction

Aortic Coarctation (AoC) is among the most prevalent congenital heart diseases (CHD). It accounts for approximately 36 (range 29–49) per 100,000 live births (1) and constitutes 5%–8% of all structural congenital cardiac lesions (2, 3). This condition occurs more frequently in males than females, with a ratio of 3:1 (2), and is frequently associated with lesions as bicuspid aortic valve, perimembranous ventricular septal defect, supra or sub-valvular aortic stenosis and more other conditions (4). Syndromic patients, particularly those with Turner syndrome, exhibit a higher incidence of AoC (4).

**Figure 1:**
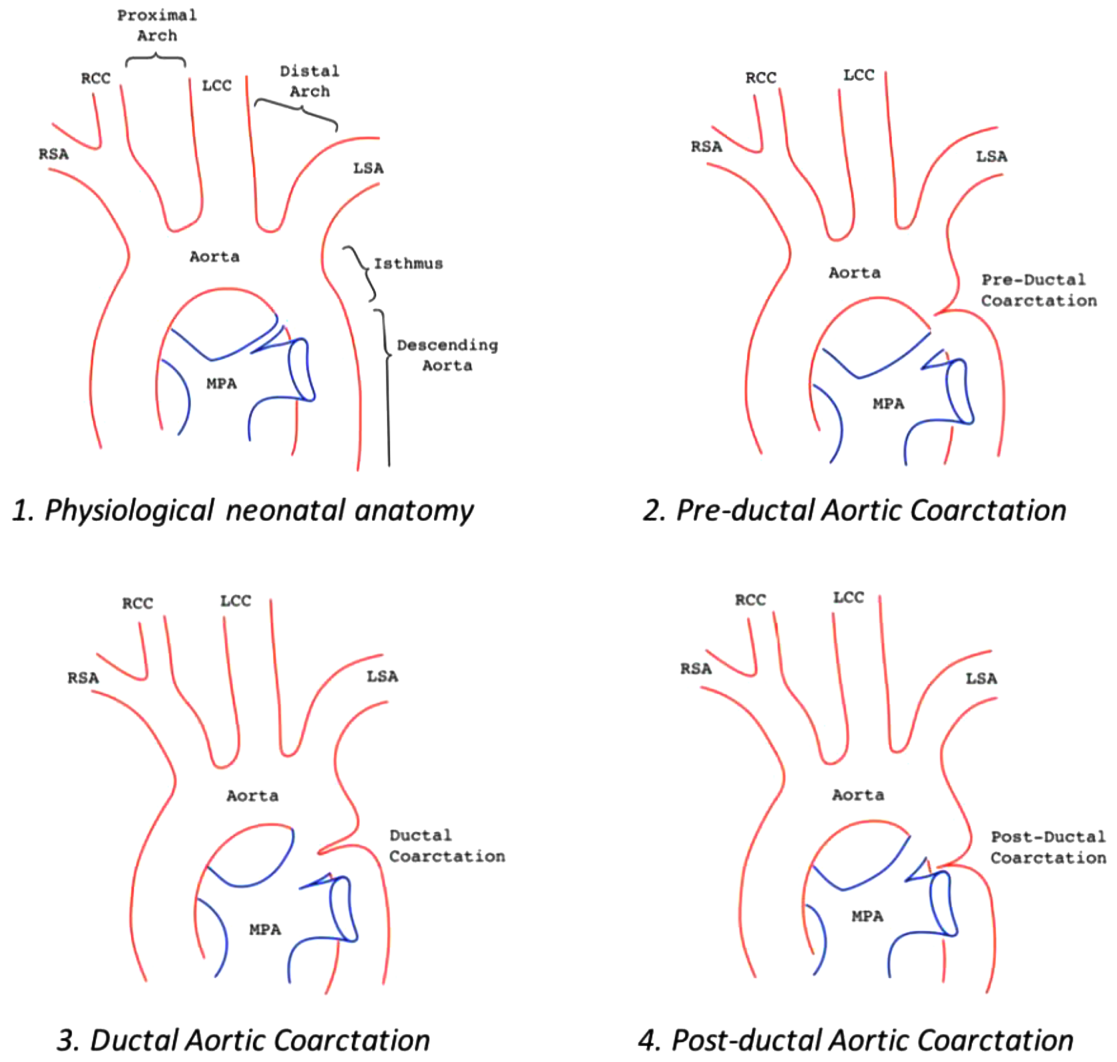
Aortic arch anatomy in different settings: 1. Normal aortic arch; 2. Pre-ductal aortic coarctation, PDA is above the stenosis; 3. Juxta-ductal aortic coarctation, the PDA is in front of the posterior shelf; 4. Post-ductal coarctation, a pressure gradient can be seen despite the PDA.

AoC is typically located at the aortic isthmus, just below the left subclavian artery, near the origin of the arterial duct. Less commonly, the narrowing may occur in the transverse aortic arch, between the left carotid artery and the left subclavian artery. Occasionally, the coarctation can be found distally in the thoracic aorta, between the arterial duct and the diaphragm (Figure 1)*.* A rare variant of AoC involves the persistent 5th aortic arch, with only a few cases reported in the literature (5–7). This condition is suspected when the narrowest point is located on the anterior aortic arch, between the innominate artery and left carotid artery.

Surgical or percutaneous correction is usually effective and provides long-term survival with an excellent quality of life post-repair. Recent studies have shown that surgery for isolated AoC is successful in 97% of patients when performed within the first year of life (8). When diagnosed at school age, percutaneous treatment demonstrates outcomes comparable to surgery (9, 10). However, AoC patients experience a higher prevalence of arterial hypertension despite successful correction. Re-coarctation occurs in approximately 5% of patients after surgery, and stent re-dilatation is often required in adolescents and young adults when the initial percutaneous procedure is performed in patients weighing less that 30 kg (11, 12). Cerebrovascular events, though rare, are a potentially life-threatening problem, due to the higher prevalence of cerebrovascular malformations in these patients (13, 14). Sometimes, very late AoC presentation might still occur, in particular in patients living or coming from geographical areas with poor health systems. Thus, even with effective AoC repair, these patients may have reduced life expectancy, increased morbidity and mortality rates, and an accelerated decline after the third decade (15–17) compared to the general population.

The aim of this review is to assess the impact of arterial hypertension in patients undergoing AoC correction and to present the current state of the art regarding the management of hypertension in this unique patient cohort.

## Diagnosis and grading of aortic coarctation

The prenatal diagnosis of aortic coarctation (AoC) remains a challenge. Despite the widespread use of fetal echocardiography in developed countries, diagnostic accuracy ranges from 48%–94% (18). Fetal cardiovascular magnetic resonance (CMR) may enhance the prediction of AoC. However, when the lesion is confined to the ductal region and not associated with aortic arch hypoplasia, postnatal monitoring is essential to confirm the diagnosis once the arterial duct get closed. Despite these limitations, prenatal diagnosis (or suspicion) improves neonatal outcomes (4, 19), by allowing for planned delivery in or near a center equipped with neonatal intensive care and pediatric cardiac surgery services. Several studies have demonstrated that timely treatment and a surgery, performed before the onset of cardiogenic shock, significantly impact both on short- and long-term outcomes (20, 21).

Despite advances, late diagnosis and management of AoC remains a current problem even in developed countries, too. The clinical presentation and age at diagnosis vary significantly, depending on the degree of aortic narrowing, potential association with other cardiac malformations or congenital syndromes, and the extent of collateral vessels development between brachiocephalic arteries and intercostal vessels.

Postnatal diagnosis typically occurs between 5 and 30 days of life, often with the patient presenting with severe cardiogenic shock. Nevertheless, AoC remains a major cause of perinatal mortality worldwide.

In cases where the closure of the ductus arteriosus is slower, the AoC is not critical and collateral circulation has developed, symptoms may be more blurred, allowing the patient to reach adulthood (1). In these patients, diagnosis often occurs following the detection of long-term complications such as hypertension, coronary artery disease, heart failure, or during a diagnostic work-up for unrelated reasons (e.g., sports participation, non-cardiac surgery, etc) (22–24).

## Treatment for AoC

Untreated AoC has a poor prognosis. Historical data indicate an average age of death at 34 years, with a 75% mortality rate by age 43 (25).

The first successful AoC repair was performed in 1944 by the Swedish surgeon Clarence Crafoord, who carried out an end-to-end anastomosis of the aorta on two patients, aged 12 and 27 years (26). Since then, several surgical approaches have been to face complex aortic arch anatomies. In 1982, percutaneous approach became available for AoC treatment (27). Despite surgery remains the primary treatment, balloon dilatation may be considered in high-risk situations (e.g., extremely low birth weight, cardiogenic shock, etc) as a bridge to surgery or as a rescue procedure in case of post-surgical restenosis.

AoC stenting, first introduced in 1991 as a rescue procedure (28), has since been refined for use in both native and recurrent AoC. The advent of smaller, more effective devices with high radial force and appropriate over-expansion capabilities has progressively established stenting as the first-line treatment in patients weighing more than 20 kg.

### Surgery

The first-line surgical approach for isolated aortic coarctation is currently the extended end-to-end anastomosis via a left posterolateral thoracotomy, as it avoids the use of patches or allografts and effectively addresses distal aortic arch hypoplasia. Alternative techniques, such as aortoplasty with patch, subclavian flap aortoplasty, and extra-anatomic grafts, were more common in previous decades but are now reserved for specific anatomies. In cases involving aortic arch hypoplasia, median sternotomy should be preferred to facilitate extended aortic arch reconstruction up to the first brachiocephalic vessel.

Complex anatomical cases may require more extensive reconstruction trough a sternotomic access, that currently is needed in approximately 5%–20% of AoC patients (8, 29).

Surgery is typically performed urgently once the diagnosis is confirmed. In premature or very low-birth-weight neonates, weight gain measures may sometimes be considered as palliative strategy (30, 31). However, successful primary surgical repair has been achieved in infants weighing over 1,000 g (32, 33), despite a higher rate of mid-term restenosis. Conversely, delayed diagnosis and/or repair in adulthood is associated with increased mortality (25). In standard settings, mortality and morbidity rates are low, with a 0.54% 30-day mortality (34).

Complications, including left recurrent laryngeal nerve injury, bronchial compression, early re-coarctation, and paradoxical hypertension, occur in approximately 5% of patients. Older age at repair (>20 years) and preoperative hypertension are associated with decreased survival rates (10). Patients younger than 9 years at the time of repair showed significantly lower rates of hypertension at 5–15 years of follow-up. Additionally, younger age at repair and end-to-end anastomosis correction are linked to fewer reintervention on the descending aorta.

### Balloon angioplasty (BA)

**Figure 2:**
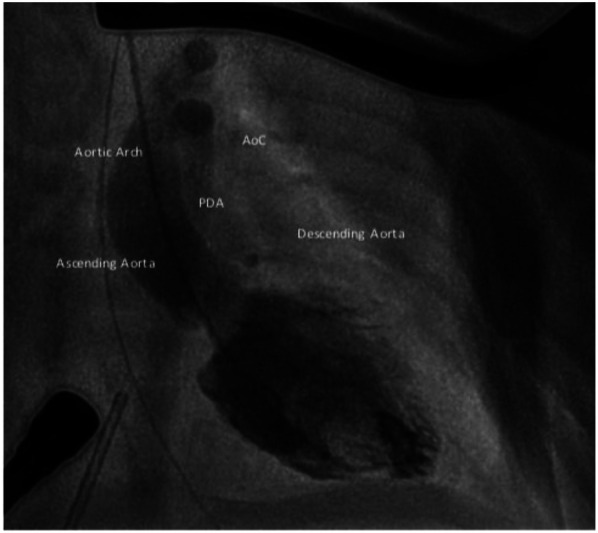
Aortic angiography after balloon dilatation from right carotid artery in 1.6 kg newborn.

The first balloon angioplasty for AoC was performed in 1982, by J. Lock (35). BA effectively reduces the pressure gradient in the short- to mid-term follow up. In cases of native AoC, it may be considered for extremely low-birth-weight infants or patients in cardiogenic shock as a bridge to surgery (36, 37) (Figure 2). However, several studies have indicated a higher risk of aneurysm formation and restenosis with isolated BA compared to surgery (4). Therefore, surgery remains the favored treatment for infants, while BA is often the first choice for managing recurrent AoC after surgical repair.

### Stent implantation

**Figure 3:**
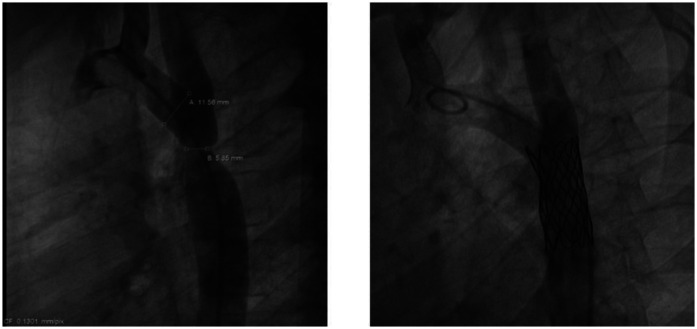
Angiographic images of aortic coarctation in an adolescent, before and after stenting.

Both the AHA/ACC and ESC Guidelines recommend percutaneous stent implantation as the first-line treatment for adolescents and adults with AoC (4, 38, 39) (Figure 3)*.* Several studies have demonstrated the high effectiveness of stenting, with lower morbidity and mortality rate compared to surgery. Unlike balloon angioplasty, stenting carries a minimal risk of aortic aneurysm and dissection (4, 40, 41).

In patients weighing between 15 and 40 kg, treatment strategies remain debated (12, 42, 43). While no definitive guidelines exist for children, stenting may be considered if the intended diameter is suitable for adult size or if the stent can be re-dilated to accommodate growth. The Cheatham-Platinum (CP) stent is the most commonly used device, although others, such as Palmaz Genesis and Andrastent (size L or XL), can also be useful, albeit with a risk of stent fracture (44, 45). Covered stents (e.g., covered CP stent, BeGraft stent, etc.) are particularly valuable in challenging cases, such as tight AoC, anatomies complicated by pseudoaneurysms, residual PDA shunts, aortic wall lesions, or aberrant vessel drainage (42).

Long-term outcomes with stents are generally excellent (7). However, late stent re-dilatation is often required when the initial procedure is performed before the age of 8, at a weight of less than 30 kg, or with a balloon diameter of less than 14 mm.

## Hypertension in operated AoC patients

Hypertension frequently complicates the long-term follow up of AoC patients, regardless of the type of correction performed. Several mechanisms contribute to the development of hypertension in AoC, including upregulation of the renin–angiotensin system, impaired vasoreactivity, aortic arch geometry abnormalities, baroreflex dysfunction, and abnormal aortic distensibility (46, 47). The pathophysiology of hypertension is still unclear and the latest hypotheses are well summarized by De Divitiis (47). Although some of these alterations might be transient or reversible, AoC patients tend to experience hypertension more frequently and at a younger age compared to the general population (48).

Diagnosing hypertension in patients with aortic coarctation can be challenging due to its variable presentation. Even after successful resolution of AoC, blood pressure readings in the left arm may be lower than in other locations, often due to hypoplasia, stenosis, or occlusion of the left subclavian artery. Furthermore, anomalies of the right subclavian artery, such as an aberrant lusory origin as the last branch of the brachiocephalic vessels, can complicate the assessment. In rare cases, it may not be possible to obtain non-invasive measurements of aortic pressure above the site of correction. These anatomical conditions can potentially mask arterial hypertension, leading to underdiagnosis or delayed treatment.

Cardiologists should carefully consider the patient's anatomical variations and remain vigilant for potential inaccuracies in non-invasive blood pressure measurements. For patients with standard anatomy, blood pressure should primarily be measured in the right arm to ensure reliable readings.

### Paradoxical hypertension

Paradoxical hypertension is often associated with AoC repair. The pathogenesis has yet to be determined, but it may be related to anatomical changes in the aorta and increased sympathetic nervous system activity: elevated plasma renin (49) and norepinephrine levels have been observed (50). This increase in norepinephrine is hypothesized to result from baroreceptor adaptation. After surgery or stent implantation, the pressure in the proximal aorta decreases, causing baroreceptors to reduce their inhibitory influence on the bulbar vasomotor centers. These centers then increase sympathetic nerve activity to compensate for the lowered proximal blood pressure, leading to increased norepinephrine release (50).

Post-surgical pain can also exacerbate hypertension, so adequate analgesic treatment is critical to mitigate this risk. Early management of postoperative hypertension is essential to reduce the risk of stroke, hemorrhage, and end-organ dysfunction.

### Chronic hypertension

Chronic hypertension is present in 20%–70% of AoC patients (2, 51), with its prevalence influenced by several factors. As expected, age is the most significant determinant. Studies have shown that the age at repair is the strongest predictor of long-term hypertension, independent of anatomical normalization (52, 53). This point might be due to several factors. First, early correction of AoC may limit exposure to hypertension and vascular wall stress (20, 54). Second, an effective surgical repair may prevent or avoid aortic arch hypoplasia. When surgical correction is performed above 15–20 kg, it becomes increasingly difficult to fully isolate the aortic arch, and the extent of surgical resection is larger, leading to significant stretching of the aortic segments (20). Similarly, percutaneous treatment of native AoC typically involves placing a stent distal to left subclavian artery, resulting in a stiffer, less elastic segment (21).

AoC patients have been shown to exhibit increased arterial stiffness and impaired flow-mediated arterial dilatation, suggesting a generalized impairment of large vessel function that coexists with AoC (54, 55). This impairment is more pronounced in patients with bicuspid aortic valve (56).

Pediatric obesity is also a known risk factor for hypertension (4), and its association with AoC leads to higher blood pressure and an increased risk of left ventricular hypertrophy in adolescents and young adults (57). On the other hand, different surgical techniques do not appear to significantly influence long-term blood pressure outcomes (58, 59).

Finally, residual aortic coarctation, palliative surgical strategies by using extra-anatomic conduits, coarctation repair by patch, complex aortic arch anatomies, and association to complex congenital heart diseases are additional risk factors for chronic hypertension (8, 29, 48, 60).

### Masked hypertension

Despite the most effective surgical or interventional treatment, hypertension remains more common in AoC patients. In young patients with repaired AoC, masked hypertension (MH) may develop early and is sometimes associated with abnormal left ventricular structural and functional changes. These patients may have increased LV mass despite normal office blood pressure readings. In such cases, 24 h ambulatory blood pressure monitoring (ABPM) can help unmask this condition (48). The earliest sign of MH in ABPM is a non-dipper profile, characterized by the absence of a normal nocturnal decline in systolic and diastolic blood pressure. Eventually, daytime hypertension may also develop. Early diagnosis of masked hypertension enables prompt treatment: once residual AoC is excluded, treatment should be considered (see above) to prevent left ventricular systolic and diastolic dysfunction. Untreated hypertension is, in fact, a major determinant of long-term morbidity and mortality in these patients.

### Exercise induced hypertension (EIH)

In AoC patients, hypertension can be exercise-induced. Blood pressure may increase during sport or physical activity, with the magnitude depending on the kind and intensity of exercise. Aerobic sports, such as cycling, running, and swimming, typically cause mild increase in blood pressure. In contrast, isometric and anaerobic sports (e.g., diving, weightlifting, shot put, etc) can lead to significant rises in both systolic and diastolic blood pressure. Finally, several sports, such as artistic and rhythmic gymnastics, volleyball, water polo and basketball may involve both types of exercise.

In clinical practice, exercise-induced hypertension is assessed using cycle ergometer or treadmill tests. While standard cut-off points exist for defining arterial hypertension in adults, well-defined prognostic standards for pediatric populations are still lacking. Specifically, in children, ranges vary based on several parameters, including sex, weight, and age (61). A study by Luitingh et al. demonstrated that patients with a peak exercise systolic blood pressure (SBP) exceeding 190 mmHg were consistently hypertensive ABPM and suggested that this threshold may be lower in younger population (62).

This condition occurs in up to one-third of the normotensive AoC patients (63, 64) and is considered an early indicator of hypertension, associated with a higher risk of developing chronic hypertension over the mid-term (65–67). As a result, exercise testing is routinely used to screen AoC patients from adolescence onwards. EIH patients are at increased risk for cardiovascular events and more pronounced LV remodeling (64) compared to normotensive patients. In adults with repaired AoC, EIH testing can also provide prognostic information and assess the efficacy of pharmacological treatment (64).

## Diagnostic workup

### Follow-up and monitoring after AoC correction

After correction, patients with AoC should undergo regular screening to assess their risk of developing hypertension. Follow-up should occur at least annually and include a clinical evaluation, ECG and echocardiography.

### Clinical evaluation

A comprehensive clinical evaluation for patients with AoC should include the assessment of radio-femoral delay, measurement of BMI, body surface area (BSA), and blood pressure in all four extremities at routine visit. This approach ensures identification of blood pressure discrepancies, a key diagnostic feature in this population. Blood pressure measurements should be taken in both arms and one or both legs, with leg measurements performed at least once during follow-up, particularly if a percutaneous procedure via the femoral artery was conducted. Z-scores for office blood pressure and ABPM are available based on sex, age and BSA. Overweight patients should be encouraged to lose weight and increase physical activity to enhance blood pressure control.

### ECG

ECG is recommended for all patients with hypertension (68) and may be useful to identify patterns of myocardial hypertrophy (47). In adults, Sokolov's Index is frequently used to suspect left ventricular myocardial hypertrophy. In pediatric age, it cannot be used because of the high risk of false positive findings. Thus, in those patients, R wave in D-II higher than 20 mV is generally used as cut-off parameter for left ventricular hypertrophy.

### Echocardiography

Echocardiographic is recommended in patients with hypertension and ECG abnormalities (68). Assessment should include evaluation of systolic and diastolic function, the aortic flow pathway, and the presence of pressure gradients at the aortic valve, transverse arch and isthmus. Additionally, pulsed wave Doppler in the abdominal aorta can help assess blood flow propagation and elastic recoil (4, 69, 70). Echocardiography is a valuable tool for assessing aortic re-coarctation, commonly identifying a peak-to-peak gradient exceeding 20 mmHg, radiological evidence of narrowing with significant collateral flow, and left ventricular hypertrophy (70). However, the correlation between echo-derived isthmic gradient and invasive pressure gradient can be weak. Several factors can impact on echo velocities: the length of the stenosis, the associated aortic arch hypoplasia, the presence of hypertrophic collateral vessels bypassing the stenosis, impaired left ventricular function, the association with other congenital heart disease (in particular ventricular septal defect or patent ductus arteriosus), or sub-optimal alignment between probe and flow. Peak velocity (>2,5 m/s), mean gradient (>20 mmHg), V2-V1 peak gradient (>20 mmHg) and the presence of a diastolic flow tail were proposed as echo marker of AoC (71). On the other hand, the evidence of a demodulated abdominal aortic flow pattern is often the strongest predictor of clinically significant AoC. Anyhow, the utility of echocardiography is constrained by operator dependency, variability in acoustic windows, and limitations in visualizing extracardiac structures and collateral circulation. Consequently, advanced imaging modalities may be required for comprehensive evaluation in certain cases (4, 70).

### Functional assessment of the LV

Functional assessment of left ventricle includes evaluating wall thickness and calculating LV mass (70). Patients with AoC often develop LV pressure overload, leading to compensatory hypertrophy and, in some cases, myocardial fibrosis (70, 72). Despite successful repair, LV function frequently remains suboptimal, underscoring the importance of monitoring LV performance during long-term follow-up of CoA patients (72). Chronic pressure overload in CoA patients also significantly affects the left atrium (LA), leading to structural remodeling, fibrosis, and impaired function. LA strain imaging offers a valuable tool for evaluating both LA and LV performance throughout the cardiac cycle (73). Studies have shown evidence of LA dysfunction and LV diastolic dysfunction (LVDD) in CoA patients (74), however it is unknown whether these indices can be used for prognostication in this court of patients (75–77). A study showed that LA strain might show a potential clinical application, on the other side LV diastolic dysfunction is affected by too many factors (74).

In overweight and obese patients (78), LV mass should be indexed either by BSA or by height raised to the power of 2.7 (79). Most echocardiography software and reporting systems automatically calculate LV mass index based on BSA, which is generally sufficient. However, in obese patients, an increased BSA may lead to an underestimation of LV mass index, so using height may provide a more reliable measure.

### Speckle tracking and myocardial function

Speckle tracking echocardiography is a sensitive tool for detecting subclinical sub-endocardial wall stress, which may be caused by residual AoC or newly onset of hypertension. Reduced global longitudinal strain values may warrant further anatomical evaluation and assessment of hypertensive status (80).

Even in patients with successful AoC repair and no hypertension, impaired longitudinal deformation properties have been observed (78). The degree of impairment correlates with age at repair and aortic stiffness (78).

Although early repair may delay hypertension onset, it cannot prevent the structural and functional abnormalities in the aorta that negatively affect myocardial deformation (78). In hypertensive patients with apparently normal systolic and diastolic function, applying the strain-time index (STI), can reveal preclinical LV systolic dysfunction (81, 82).

### Ambulatory blood pressure monitoring (ABPM)

ABPM is a useful, non-invasive, and well-tolerated tool for diagnosing masked hypertension (83). Typically, the device records 50–70 blood pressure measurements, providing mean values and standard deviation for 24 h, daytime and nighttime periods. The report also lists the number of values outside the normal range for systolic and diastolic pressures. Standard settings apply to adult patients. Thus, before starting with data analysis, cut-off values should be tailored according to age, sex and BSA percentiles. Normally, mean values should remain below the upper limit identified, with less than 20% of readings above the threshold. At night, blood pressure should decrease by at least 10% (84). The absence of this nocturnal reduction is termed a “non-dipper profile” (85). It is important to note that the device's default nighttime settings should be adjusted based on the patient's sleep diary. ABPM is also valuable for monitoring the effectiveness of hypertension treatment, allowing clinicians to adjust doses and timings accordingly (84, 86).

### Exercise testing

Exercise testing is especially useful for identifying exercise-induced hypertension (64, 65). As mentioned above, BP should be measured in the right arm. An abnormal exercise BP profile may be due to several factors, such as mild or masked residual AoC, loss in aortic elasticity, idiopathic reasons. A bicuspid aortic valve can also increase the risk of idiopathic hypertension. Additionally, aortic valve stenosis may obscure or generate Doppler artifacts, making it difficult to assess isthmic flow and gradients. To overcome these limitations, an echocardiographic stress monitoring of the aortic arch may help clarify the diagnosis in these cases. Mild to moderate AoC can present with phasic isthmic flow and preserved abdominal aorta pulsed wave shape. With increased cardiac output, the Doppler profile might change. Aortic stenosis typically results in a systolic-only flow, whereas residual AoC may show a diastolic flow tail in both the arch and the abdominal aorta. Patients with moderate residual AoC and exercise-induced hypertension or aortic valve regurgitation may benefit from treatment of the residual lesion. A recent study has shown that exercise blood pressure may provide prognostic information and assess antihypertensive therapy efficacy in adults with repaired CoA (64, 66, 67).

### Anatomical assessment of the aortic arch

**Figure 4:**
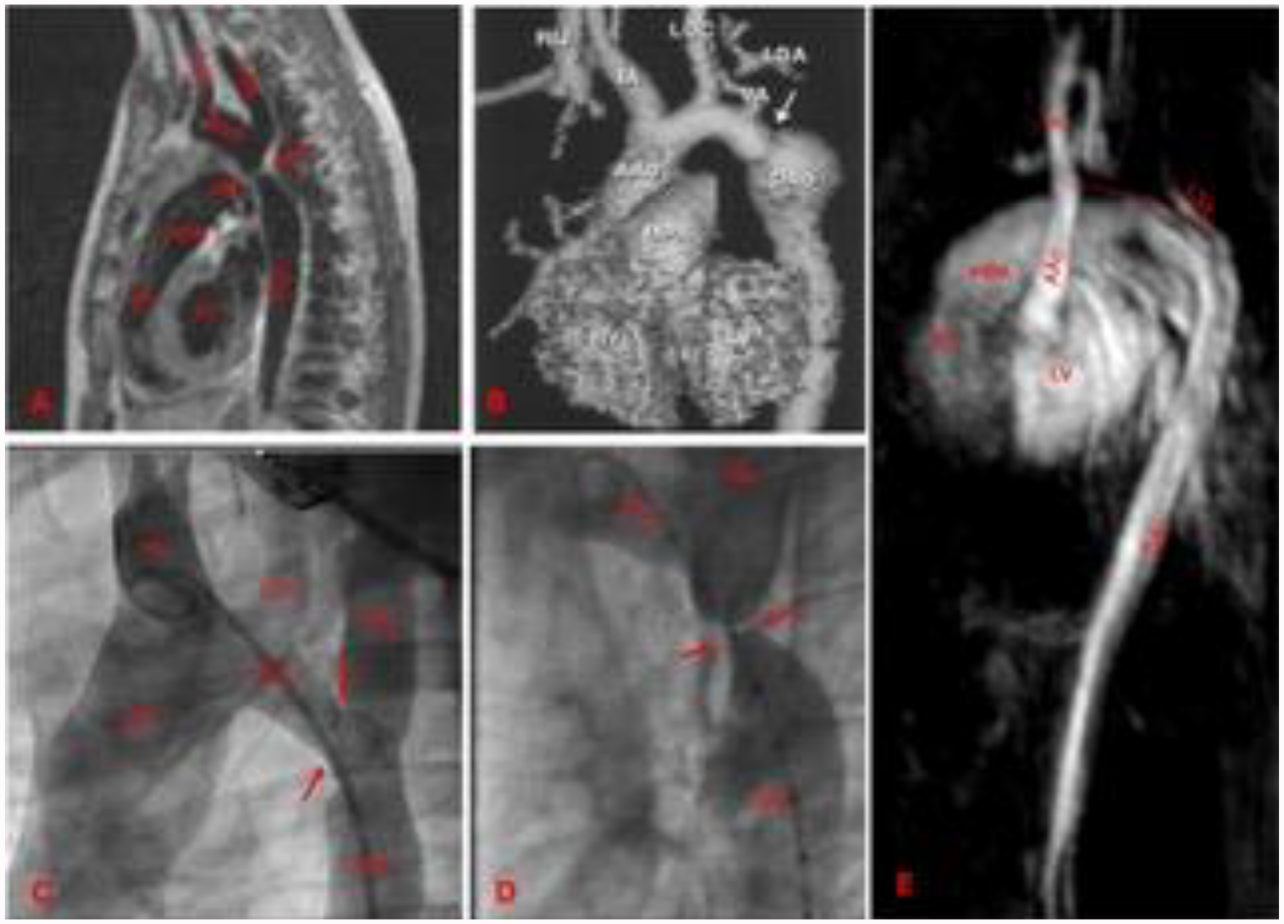
Different aortic coarctation morphologies by CMR or angiographies. **(A)** Membrane shaped by CMR, there is short segment involved. **(B)** CMR-based aortic arch 3D model. Post-surgical aortic coarctation. There is a distal arch re-stenosis, associated with a post-stenotic aneurism of the aortic wall. **(C)** Angiography of a transverse aortic arch hypoplasia associated to aortic coarctation. **(D)** Angiography of an aortic coarctation with sub-atresia. The lumen in the narrowest tract is as large as the size of the catheter (5 Fr). **(E)** CMR view of a neonatal severe form of type 2 interrupted aortic arch. The dotted red line indicates the atretic tract. Arrows indicate the stenosis. LV, left ventricle; RV, right ventricle; MPA, main pulmonary artery; IA, innominate artery; LCC, left common carotid; LSA, left subclavian artery; AAo, ascending aorta; Arch, aortic arch; Dao, descending aorta.

Anatomic assessment of the aortic arch involves either CT scan or angio-CMR. CT scan with angiographic sequences provides detailed information about the aorta, supra-aortic vessels, and thoracic collaterals (Figure 4)*.* It is the gold standard for detecting pseudoaneurysms, intimal tears and dissections (87). When performed with ECG gating and end-systolic acquisitions, CT can also aid in procedural planning (88, 89). However, the use of iodine contrast and radiation exposure limits the repeatability of this exam.

Angio-CMR provides high-quality images of the aorta, comparable to those of a CT scan in both native and surgically treated AoC. If limited to black-blood acquisitions, contrast media can be avoided. However, gadolinium contrast enhances anatomical details and provides important information about gradients (88) and abdominal blood flow. Like echocardiography, systolic peak and diastolic flow tails are the key indicators of residual AoC. While angio-CMR is more time-consuming, operator-dependent, more expensive, and may require sedation in infants and children (70), it is more repeatable than CT, especially when performed without contrast. Unfortunately, its diagnostic accuracy worsen significantly when metallic device are present in the aorta (e.g., aortic stents, ductus arteriosus devices, etc) or nearby (e.g., mechanical valves, pacemaker, orthopedic prostheses, etc). In these cases, a CT scan is preferred.

### Cerebral arteries and AoC

Patients with AoC are at risk of cerebral artery anomalies, particularly vascular aneurysms (13, 14). In these patients, hypertension can lead to aneurysm rupture and consequent cerebral hemorrhage. Therefore, imaging of cerebral arterial tree (*via* angio-CT or angio-CMR) might be proposed at diagnosis in AoC patients diagnosed out of neonatal age.

### Atherogenic effects

Hypertension is a strong determinant of carotid intima-media thickness (CIMT), which is a predictor of atherosclerosis. Consequently, the effect of statins on patients with repaired AoC has been studied. In the study of Luijendijk P et al. (90) it was confirmed that hypertensive patients with repaired AoC exhibited significant CIMT progression. However, in this study, atorvastatin treatment was not effective in reducing these complications, despite a marked reduction in serum total cholesterol and LDL levels. Conversely, the study of Brili et al. (91) demonstrated significant improvements in endothelial function and decreased circulating levels of pro-inflammatory cytokines in patients with repaired AoC.

During long-term follow-up after AoC repair, cardiovascular risk increases due to an endothelial dysfunction and elevated expression of inflammatory proteins. The effect of ramipril's (an ACE inhibitor) was studied in these patient group and was shown to be effective in improving endothelial function and reducing the expression of proatherogenic inflammatory cytokines and adhesions molecules (92).

## Therapies for hypertension in repaired-AoC

Hypertension treatment involves both peri-operative care and long term follow-up. In the first case, hypertension is a transient event, resulting from the relief of stenosis and altered stimuli on renal and carotid pressure receptors. Chronic hypertension, on the other hand, is a more insidious condition, arising from a less elastic aortic wall, which predisposes patients to an early onset of hypertension.

### Post-surgical hypertension

Intravenous drugs are usually preferred in the intensive care unit. Hypertension is quite common after surgery, potentially due to neuro-autonomic changes and the pain associated with the procedure. Achieving adequate BP control is essential to reduce the risk of cerebral bleeding and suture dehiscence. Additionally, lowering BP reduces afterload, which can be beneficial in patients with preoperative left ventricular dysfunction. Thus, antihypertensive might also have an inotrope-sparing effect.

*Sodium nitroprusside (SNP)* is typically the first choice for treating hypertension following aortic coarctation repair (93). Its effect results from its breakdown into nitric oxide (NO), which exerts a potent vasodilatory effect on arterioles. The action is focused on arterial vessels, with no impact on inotropy. Due to its very short half-life, SNP can be easily managed by adjusting the dose. However, SNP promotes the development of free radicals, so its use beyond 48 h might result toxic for the patient.

*Esmolol* is a selective beta-1 receptor blocker with rapid onset (within seconds), rapid peak effect, and very short duration of action, degraded by esterases in the cytosol of red blood cells. These characteristics offer several advantages over propranolol in the treatment of paradoxical hypertension after AoC repair (94). In a study comparing esmolol to sodium nitroprusside, esmolol was found wo be effective in treating paradoxical hypertension, either as monotherapy or in combination (95), with an excellent safety profile (94). Like SNP, Esmolol doses can be easily adjusted to tailor the effect for the patient. However, like other B-1 blockers, Esmolol impacts cardiac function by reducing heart rate, inotropy, and oxygen consumption. Therefore, its use should be approached cautiously in patients with cardiogenic shock.

*Labetalol* is a non-selective, competitive beta-adrenergic (B1 and B2) blocker and a selective alpha1-adrenergic antagonist, with a rapid onset and peak effect, and a half-life of 3–5 h. Unlike Esmolol, Labetalol has a longer duration of action, making dose adjustments more difficult. Retrospective study have indicated that it is a safe single-agent therapy for treating hypertension post-coarctectomy, with the added advantage of easy transition from intravenous to oral administration (96). However, this therapy has a negative association with ductus-dependent circulation (96).

*Dexmedetomidine* is an intravenous analgo-sedative used both intraoperatively and postoperatively. It is a highly selective alpha-2 agonist, a drug that exerts multiple effects. Dexmedetomidine reduces central sympathetic output, inhibits the release of epinephrine, norepinephrine and renin release, thereby lowering arterial blood pressure (97, 98). In conclusion, its ancillary effects on the cardiovascular system, combined with its primary sedative and analgesic effects, make this drug ideal for postoperative care of patients with AoC in intensive care unit (97, 99). A recent study demonstrated that dexmedetomidine is safe and that it reduces the incidence and severity of paradoxical hypertension, as well as the need for antihypertensive medications in patients undergoing aortic coarctation repair (100).

After the first 48 h, patients can often be weaned off intravenous antihypertensive drugs. Once discharged from intensive care unit, the patient may either remain off therapy or transition to oral therapy. The choice is based on the pre-surgical clinical condition, BP values, and patient's age. Neonates with a pre-natal diagnosis of AoC can be treated promptly with adequate LV function. In these patients, mid-term oral treatment is usually unnecessary. Patients without a prenatal diagnosis often present for surgery in poor clinical condition, with low cardiac output, biventricular dysfunction, metabolic acidosis, and oligo-anuria. In these cases, mid-term oral therapy should be considered to promote the left ventricle reverse remodeling. In adult patients, hypertension may persist for several weeks following AoC correction; therefore, mid-term therapy is also recommended for these patients.

### Oral therapies for (chronic) hypertension

In pediatric patients (including both children and adolescents), there is currently no general consensus on the management of arterial hypertension. In the normal population the most recent guidelines agree on initiating the treatment with non-pharmacological interventions, focusing on improving adherence to a healthy lifestyle, including reducing the intake of salt-rich foods (101).

However, this approach is not recommended in patients with AoC, as hypertension in these cases is caused by a structural issue. In this cohort of patients, pharmacological therapy is indicated.

The range of drugs available for chronic hypertension is extensive. However, the options available for pediatric patients are much more limited. Similarly, data on hypertension in CHD patients, including AoC, is restricted to only a few drug classes.

### Beta-blockers (BB)

Beta-blockers (Table 1) are currently considered the first-line therapy for AoC. Numerous studies have demonstrated the safety and efficacy profile of beta-blockers in patients with AoC patients (94, 96, 102). The utility of beta-blockers is not limited to hypertension management; several studies suggest their use in preventing aortic dilatation and ascending aorta aneurisms, particularly when AoC is associated to bicuspid aortic valve. Beta-blocker are classically categorized into β1-selective and non-selective.

**Table 1:** Beta blockers used in clinical practice.

Class	Drug	Characteristics	Dosage	Controindications	Side effects
Beta-blockers BBs	Propranolol	Non selective	*Children*	Asthma, congestive heart failure. Beware hypoglycemia in infants and diabetics	Hypotension, syncope, bronchospasm, nausea and vomiting, hypoglycemia, lethargy and depression, hearth block
		1st pass hepatic metabolism (pharmacologically active metabolites)	PO: 0.5–1 mg/kg/24 h each 12 h-6 h.		
			Increase to 2–4 mg/kg/24 h.		
			Max dose 8 mg/kg/24 h		
		High lipophilicity	*Adults*		
		Dosable (beta blockade 25–150 ng/ml)	PO: 10–25 mg/dose each 8 h-6 h		
		Renal elimination			
	Nadolol	Non selective	*Children*	Asthma, congestive heart failure. Beware hypoglycemia in infants and diabetics	CNS (dizziness, tiredness, depression, tinnitus), bradycardia, bronchospasm, diarrhea, nausea and vomiting, rash
		Low lipophilicity	PO: 0.25–0.5 mg/kg/24 h;		
		Renal elimination	Adjust up or down after 5 days based on side effects, sinus rate and efficacy		
			*Adults*		
			PO: 40–240 mg/24 h		
	Atenolol	β1 selective	*Children*		CNS (dizziness, tiredness, depression), bradycardia, postural hypotension, nausea and vomiting, rash, blood dyscrasias (agranulocytosis, purpura)
		Low lipophilicity	PO: 1–2 mg/kg/dose, each 24 h		
		Renal elimination	*Adults*		
			PO: 25–100 mg/dose, 24 h for 1–2 week; may increase to 200 mg 24 h		
	Metoprolol	β1 selective	*Children >*2 yr		CNS (dizziness, tiredness, depression), bronchospasm, bradycardia, diarrhea, nausea and vomiting, abdominal pain
		High lipophilicity	PO: Initial 0.1–0.2 mg/kg/dose, each 12 h; gradually increase to 1–3 mg/kg/24 h		
		Hepatic elimination	*Adults*		
			PO: initially 100 mg/24 h, each 24 h-8 h		
			Usual dose 100–450 mg/24 h		

First-generation beta-blockers, such as propranolol, nadolol, timolol, sotalol and pindolol, block both *β*1 and *β*2 receptors. Consequently, this group affects cardiomyocytes, smooth muscle cells in blood vessels, and the lungs, with bronchoconstriction as a potential side effect. Thus, these drugs are no longer used to treat hypertension.

Second-generation beta-blockers, including metoprolol, acebutolol, bisoprolol, esmolol, betaxolol, and acebutolol, are β1 selective. This group has been extensively studied for heart failure and heart rate control. They have significantly lower impact on bronchoconstriction and peripheral vasodilatation.

Pindolol, penbutolol, and acebutolol differ from other beta-blockers due to their intrinsic sympathomimetic activity (ISA), which can increase blood pressure and heart rate. This class of Beta-Blockers has a smaller effect on reducing resting cardiac output and resting heart rate compared with other classes.

Third-generation Beta-blockers include labetalol and carvedilol, which block both β- and α1-adrenergic receptors, creating a synergistic effect that induces vasodilation and to reduces blood pressure.

Beta-blockers can also be classified as lipophilic and hydrophilic. The clinical significance of this classification relates to the volume of distribution and the drug's effect on the brain. Lipophilic Beta-blockers can cross the blood-brain barrier and exert additional effects on the central nervous system, therefore, the use of this class of drugs should depend on the risk of adding depressive symptoms.

Propranolol was the first beta-blocker available on the market. It was effectively used in the perioperative period for coarctation repair, where it was shown to significantly reduce systolic and diastolic blood pressure as well as plasma renin activity (103). Its role has been evaluated as a prophylactic therapy for the prevention of paradoxical hypertension after AoC repair (103, 104). Propranolol has a relatively short half-life, requiring administration 3–4 times per day.

Atenolol is a non-selective beta-blocker that can be administrated once or twice per day. Over the past two decades, several studies have demonstrate that Atenolol may be considered a first-line treatment for AoC.

The most common side effects of BB include bradycardia, bronchospasm, asthma, Raynaud's disease, and hypoglycemia in diabetics.

### Calcium channel blockers (CCBs)

Calcium channel blockers (CCBs) (Table 2) play a pivotal role in the management of AoC. This class of drugs is divided in two categories: dihydropyridines and non-dihydropyridines. The first group acts selectively on peripheral arteries, causing arteriolar vasodilation and an effective reduction in blood pressure. Non-dihydropyridines also act on cardiomyocytes, primarily suppressing sympathetic stimuli, thereby decreasing heart rate, blood pressure, inotropy and dromotropy.

**Table 2:** Calcium channel blockers currently used in chronic hypertension.

Class	Drug	Characteristics	Dosage	Controindications	Side effects
Calcium channel blockers CCBs	Amlodipine	Dihydropyridines	*Children*		Edema, dizziness, flushing, palpitation, headache, fatigue, nausea, abdominal pain, somnolence
		(3rd gen)	PO: initial 0.1 mg/kg/dose each 24 h-12 h may increase gradually to a max of 0.6 mg/kg/24 h		
		L-type, N-type channels			
		Hepatic metabolism			
		Renal elimination 60%, hepatic elimination 20–25%	*Adults*		
			PO: 5–10 mg/dose each 24 h		
			(max 10 mg/24 h)		
	Nicardipine	Dihydropyridines	*Children*	Absolute	Edema, dizziness, flushing, palpitation, headache, fatigue, nausea, abdominal pain, somnolence
		(2nd gen)	PO: 0.4–0.8 mg/kg each 8 h	Pregnancy	
		Hepatic metabolism	*Adult*	Lactation	
		Hepatic elimination 70%	PO: 20–40 mg each 8 h	Absolute	
		Renal elimination 30%		Hepatic insufficiency	
				Renal failure	

CCBs are typically used as second-line treatment in pediatric patients due to the risk of hypotension. They are often considered when BBs are insufficient to achieve adequate blood pressure control. Dihydropyridine CCBs are commonly used to treat postoperative hypertension in adults are nifedipine, amlodipine and nicardipine are the almost known molecule. They have a rapid onset and peak effect, increase cardiac output by enhancing venous return, and reduce oxygen consumption by lowering afterload (105). The half-life of CCBs is usually short or very short, making them particularly effective in case of hypertensive crises. However, for chronic use, controlled-released formulations are needed to stabilize plasma concentration and reduce the frequency of daily administration.

The most common side effects of CCBs include flushing, headache, peripheral edema, dizziness, and paradoxical hypotension.

### Angiotensin converter enzyme inhibitors (ACE-I) and angiotensin receptor blockers (ARB)

ACE-I (Table 3) are a common first-line treatment for hypertension at any age. They induce arteriolar vasodilatation. Due to their large extensive use in pediatric and neonatal heart failure, wide therapeutic range and low risk of adverse effects, ACE-Is are increasingly replacing BB as first-line treatment for hypertension. Several ACE-I molecules are available, differing in onset and half-life. Captopril was the first ACE-I introduced, with a rapid onset and short half-life. Requiring administration three of four times daily for complete coverage. Enalapril has a slightly longer half-life and is administrated twice daily, while Ramipril, Lisinopril, and Perindopril allow for once-daily administration. Unlike BB, ACE-Is mainly differ in terms of half-life and time to peak dose.

**Table 3:** Angiotensin converting enzyme inhibitors to treat hypertension.

Class	Drug	Characteristics	Dosage	Controindications	Side effects
Angiotensin converting enzyme inhibitors ACE-i	Enalapril	Pro-drug (active metabolite enalaprilat)	*Children*	*Absolute*	Hypotension, dizziness, fatigue, headache, rash, diminishing taste, neutropenia, hyperkalemia, chronic cough
			PO: 0.1 mg/kg/dose each 24 h-12 h may increase over 2 weeks to a max of 0.5 mg/kg/24 h	Angioedema	
		Renal elimination (60% enalapril, 40% enalaprilat)		Pregnancy	
				Lactation	*Fetal risk if given during 2nd and 3rd trimesters*
			*Adults*		
			PO: 2.5 mg/dose each 24 h-12 h (max 10–40 mg/24 h)		
	Captopril		*Neonates*	*Absolute*	Neutropenia/agranulocytosis, proteinuria and tachycardia, rash, taste impairment, hyperkaliemia
			PO: 0.1–0.4 mg/kg/24 h, each 8 h-6 h	Angioedema	*Fetal risk if given during 2nd and 3rd trimesters*
			*Children*	Pregnancy	
			PO: Initially 0.3–0.5 mg/kg/dose, each 8 h (max 6 mg/kg/24 h, 12 h-6 h)	Lactation	
			*Adolescents and Adults*		
			PO: 12.5–25 mg/dose each 12 h-8 h		
			Increased weekly if needed by 25 mg/dose to max dose of 450 mg/24 h *(adjust dose with renal failure)*		
	Ramipril	Pro-drug (active metabolite ramiprilat)	*Children*	*Absolute*	Dry non-productive cough, dizziness, fatigue, nausea, hyperkalemia, angioedema, rarely neutropenia,
			PO: 0.05 mg/kg/24 h, may increase over 4–6 weeks to 0.1–0.2 mg/kg/24 h	Angioedema	
		Renal elimination 60%		Pregnancy	*Fetal risk if given during 2nd and 3rd trimesters*
		Hepatic elimination 40%	*Adult*	Lactation	
			PO: 2.5 mg/24 h, may increase over 4–6 weeks to 5–10 mg/24 h		
	Lisinopril	No protein binding	*Children *≥* 6 yr*	*Absolute*	Dry non-productive cough, rash, hypotension, hyperkalemia, angioedema, rarely bone marrow depression
		Renal elimination	PO: initially 0.07 mg/kg/dose 24 h (max initial dose 5 mg/24 h) may increase over 2 weeks to a max of 0.6 mg/kg/24 h or 40 mg/24 h	Angioedema	
				Pregnancy	
				Lactation	*Fetal risk if given during 2nd and 3rd trimesters*
			*Adults*		
			PO: 10 mg/dose 24 h (max 80 mg/24 h)		
	Perindopril	Pro-drug (active metabolite perindoprilat)	*Children*	Absolute	Cough, fatigue, asthenia, headache, disturbances of mood or sleep, taste impairment, epigastric discomfort, nausea, abdominal pain, rash
			PO: 0.05–0.15 mg/kg/24 h	Angioedema	
		Renal elimination	*Adults*	Pregnancy	
			PO: 2–8 mg/24 h	Lactation	*Fetal risk if given during 2nd and 3rd trimesters*

The most common side effects of ACE-Is include renal dysfunction, hyperkalemia, and cough. ACE-Is are contraindicated during pregnancy.

Angiotensin receptor blockers (ARBs) (Table 4) act by inhibiting the effects of angiotensin II at its receptor sites, thereby preventing its vasoconstrictive action and reducing sodium and water retention through modulation of renal and vascular pathways. ARBs specifically block AT1 receptors, which are found in the heart, blood vessels and kidneys. Consequently, ARBs are used to treat hypertension, heart failure and chronic kidney disease. Additionally, they can be used to prevent aortic wall dilatation in collagenopathies (e.g., Marfan Syndrome). Losartan, Valsartan, Irbesartan, Olmesartan, and Candesartan are the most commonly used ARBs. They may be considered first-line drugs in adults due to the high response rate, low incidence of adverse effects, and long-acting nature, which typically allows for once-daily dosing. In pediatric patients, ARBs are considered when ACE-Is are poorly tolerated (e.g., due to cough). Side effects include hyperkalemia, altered taste, and skin rash. Similar to ACE-Is, ARBs are contraindicated during pregnancy.

**Table 4:** Angiotensin receptor blockers to treat hypertension.

Class	Drug	Characteristics	Dosage	Controindications	Side effects
Angiotensin receptor blockers ARBs	Losartan	Pro-drug (active metabolite EXP3174)	*Children *≥* 6 yr*	Absolute	Hypotension, dizziness, nasal congestion, muscle cramps, anemia, thrombocytopenia, rash
			PO: 0.7 mg/kg/dose each 24 h-12 h (max 50 mg/24 h)	Pregnancy	
		High protein binding		Lactation	*Fetal risk if given during 2nd and 3rd trimesters*
			*Adults*	Relative	
		Hepatic metabolism	PO: 50 mg/dose 24H (max 100 mg/24 h)	Renal artery stenosis	
		Renal elimination 13–25%		Hepatic insufficiency	
		Hepatic elimination 50–60%			
	Valsartan	Renal elimination 30%	*Children*	Absolute	Dizziness, hypotension, diarrhea, joint pain, fatigue, back pain, rhinitis or sinusitis
		Hepatic elimination 70%	PO: 0.8 mg/kg/24 h	Pregnancy	
			*Adults*	Lactation	*Fetal risk if given during 2nd and 3rd trimesters*
			PO: 40–160 mg/24 h	Relative	
				Renal artery stenosis	
				Hepatic insufficiency	
	Olmesartan medoxomil	Pro-drug (active metabolite olmesartan)	*Children*	Absolute	Hypotension, dizziness, nasal congestion, muscle cramps, vomiting, diarrhea, weight loss
			PO: 0.3 mg/kg/24 h (max 10 mg), may increase every 2 week to max 0.8 mg/kg/24 h	Pregnancy	
		Hepatic metabolism		Lactation	*Fetal risk if given during 2nd and 3rd trimesters*
		Hepatic elimination 60%		Hepatic insufficiency	
		Renal elimination 40%	*Adults*	Biliary obstruction	
			PO: 10 mg/24 h, may increase to max 40 mg/24 h	Relative	
				Renal artery stenosis	
	Candesartan cilexetil	Pro-drug (enteric esterase metabolism, active metabolite candesartan)	*Children*	Absolute	Hypotension, dizziness, hyperkalemia, anemia
			PO: 0.1–0.3 mg/kg/24 h	Pregnancy	*Fetal risk if given during 2nd and 3rd trimesters*
			*Adults*	Lactation	
		Hepatic metabolism	PO: 4–16 mg/24 h	Relative	
		Renal elimination 33%		Renal artery stenosis	
		Hepatic elimination 67%			

## How to choose the most appropriate therapy in AoC patients

Currently, the first-line approach for hypertension in AoC is largely based on local protocols or physician preferences. A multicenter study (106) evaluated the prevalence of antihypertensive therapy at hospital discharge in 39 tertiary care pediatric hospitals in the USA between 2004 and 2013, encompassing a population of 1,636 patients. This study highlighted the significant variability in discharge prescription, reflecting the lack of evidence-based guidelines. The most commonly prescribed medications were: enalapril (43.3%), captopril (28.3%), atenolol (28.0%), propranolol (15.6%), lisinopril (5.1%), amlodipine (3.8%), metoprolol (2.9%), labetalol (2.7%), nifedipine (1.4%). All other medication were prescribed at less than 1%, demonstrating considerable variability in pharmacotherapy at discharge. Thus, ACE-Is and BBs are the most commonly used drugs in these patients.

There are few studies comparing the efficacy, safety and secondary outcomes, such as morbidity and mortality, of oral antihypertensive medications in this specific population. Among these, Di Salvo et al. conducted a randomized trial comparing *atenolol* and *enalapril* in the management of hypertension following AoC repair. The study concluded that, while both drugs effectively reduced SBP, only enalapril significantly reduced left ventricular mass/height (107). Therefore, ACE-Is may be considered the first-line choice for patients with hypertension without residual AoC and left ventricular hypertrophy.

Another study compared the effects of *candesartan* (an ARB) and *metoprolol* (a BB) in a small cohort of adult patients after 8 weeks of treatment (108). Metoprolol demonstrated a greater reduction in mean arterial pressure, although it was associated with an increase in plasma type B natriuretic peptide concentration (108). However, no larger studies have confirmed these results in a broader cohort or over a longer follow-up period.

A recent Cochrane review (109), which included 21 randomized clinical trials, found that data on the use of antihypertensive drugs in children remain limited. Candesartan was associated with a significant reduction in systolic and diastolic blood pressure compared to placebo, but no consistent dose-response relationship. ACE-Is demonstrated good efficacy in reducing systolic and diastolic blood pressure compared to baseline, though no consistent evidence was found across studies. BBs appeared less effective in children than in adults. Although CCBs are frequently prescribed, the evidence supporting their blood pressure-lowering efficacy is limited. In the short term, all evaluated antihypertensive drugs were considered safe.

**Figure 5:**
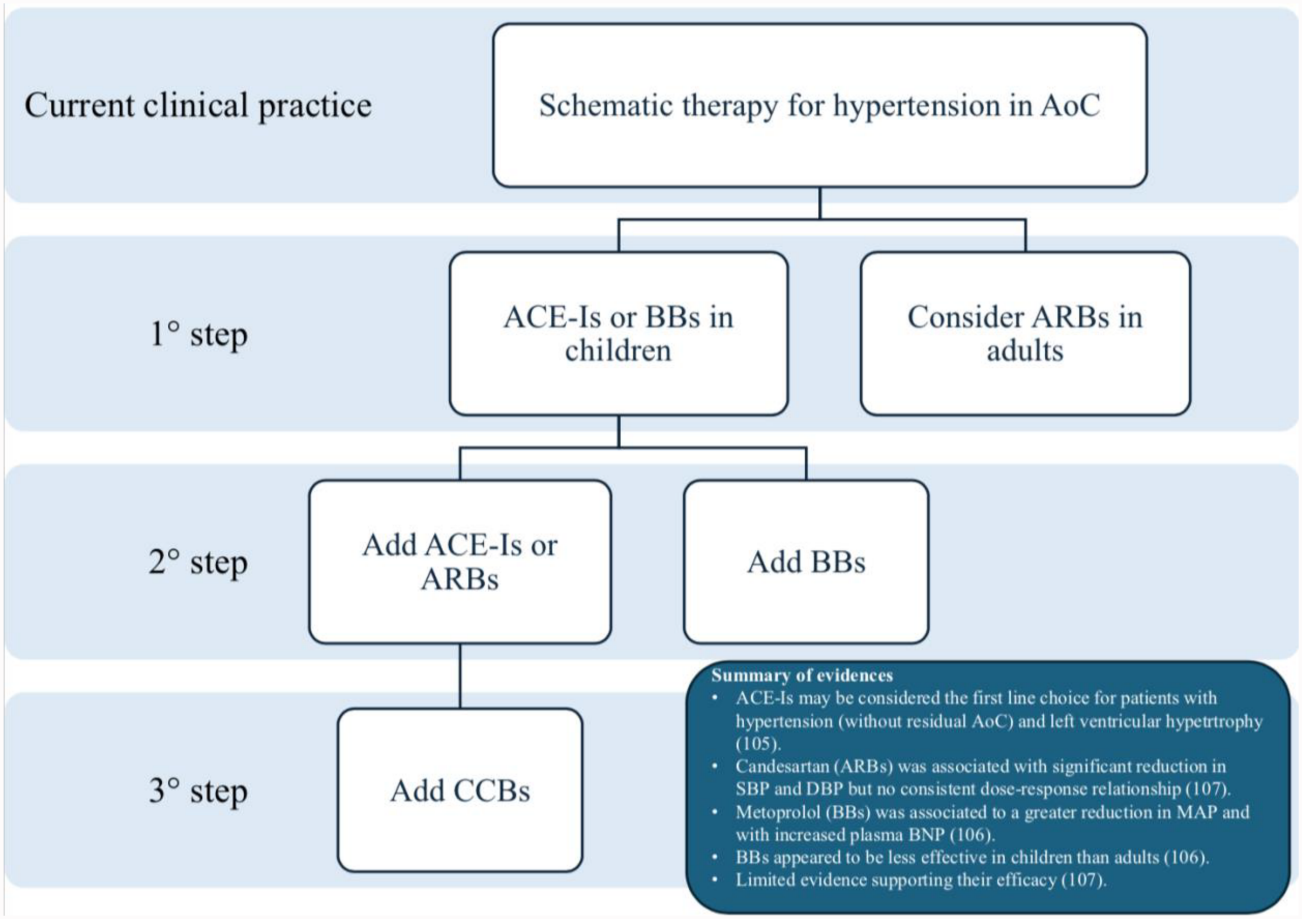
Flowchart of therapy for hypertension in AoC based on current clinical practice.

Figure 5 summarizes the most common treatment algorithms in these patients.

## Conclusion

Despite successful repair, 20%–70% of AoC patients may develop chronic hypertension during long-term follow-up. Early diagnosis can be challenging, and a multi-parametric approach is often necessary. Untreated hypertension increases the risk of cardiovascular events, atherogenic conditions, and advanced LV remodeling, leading to impaired diastolic function and LV hypertrophy. To prevent major adverse events, these patients should be followed at centers with expertise in congenital heart diseases to ensure early diagnosis and appropriate treatment.

Despite hypertension in AoC is a specific issue, not merely comparable to adult-like arterial hypertension, no dedicated guidelines or recommendations are available for these patients. Thus, treatment algorithms are often based on individual preferences or single center protocols. Our research has highlighted a predominant use of ACE inhibitors and beta-blockers as first-line options in pediatric patients, with ARBs also considered in adults. Calcium channel blockers and diuretics are commonly employed as adjunctive therapies when hypertension persists despite initial treatment.
